# REFINE-Lung implements a novel multi-arm randomised trial design to address possible immunotherapy overtreatment

**DOI:** 10.1016/S1470-2045(23)00095-5

**Published:** 2023-05-01

**Authors:** Ehsan Ghorani, Matteo Quartagno, Fiona Blackhall, Duncan C Gilbert, Mary O’Brien, Christian Ottensmeier, Elena Pizzo, James Spicer, Alex Williams, Philip Badman, Mahesh K B Parmar, Michael J Seckl, on behalf of the REFINE-Lung Investigators, Michael Seckl, Ehsan Ghorani, Joanne Evans, Pollyana D’Avila Leite, Sanjary Mistry, Emily White, Amalia Saucan, Mary O’Brien, Sanjay Popat, Charlotte Milner-Watts, Libby Hennessy, Bianca Rock, Jaishree Bhosle, Anna Minchom, Nadza Tokaca, Hazel O’Sullivan, Fiona Blackhall, Fabio Gomes, Kate Brown, Stephen Boyd, Laura Moliner Jimenez, Sarah Treece, Elisa Barter, Kerrie Cavanagh, Terri-Anne Baker, Abigail Hollingdale, Sudipta Datta, Jason Adhikaree, Rebecca Ashton, Karen Newcombe, Kathryn Tarver, Simon Ball, Jonathan Shamash, Neale O’Brien, Thi Vu, Pooja Jain, Amy Humphries, Gwendolyn Saalmink, Liz Ashford, Grant Stewart, Toby Talbot, Jane Broom, Colin Barrie, Lisa Thomson

**Affiliations:** Department of Medical Oncology, Charing Cross Gestational Trophoblastic Disease Centre, Charing Cross Hospital Campus of Imperial College London, London, UK; Institute for Clinical Trials and Methodology, University College London, London, UK; Christie National Health Service Foundation Trust, Manchester, UK; Institute for Clinical Trials and Methodology, University College London, London, UK; Royal Marsden Hospital, Imperial College London, London, UK; Institute of Systems, Molecular and Integrative Biology, University of Liverpool, Liverpool, UK; Clatterbridge Cancer Center NHS Foundation Trust, Liverpool, UK; Department of Applied Health Research, University College London, London, UK; King’s College London, Guy’s Hospital, London, UK; Imperial College Trials Unit—Cancer, Department of Surgery and Cancer, Imperial College London, London, UK; Imperial College Trials Unit—Cancer, Department of Surgery and Cancer, Imperial College London, London, UK; Institute for Clinical Trials and Methodology, University College London, London, UK; Department of Medical Oncology, Charing Cross Gestational Trophoblastic Disease Centre, Charing Cross Hospital Campus of Imperial College London, London, UK; Charing Cross Hospital; The Royal Marsden Hospital; The Christie Hospital; Peterborough City Hospital; Nottingham City Hospital; Queen’s Hospital; Leeds Hospital; Royal Cornwall Hospital; NHS Lothian

## Abstract

Increasing evidence suggests that some immunotherapy dosing regimens for patients with advanced cancer could result in overtreatment. Given the high costs of these agents, and important implications for quality of life and toxicity, new approaches are needed to identify and reduce unnecessary treatment. Conventional two-arm non-inferiority designs are inefficient in this context because they require large numbers of patients to explore a single alternative to the standard of care. Here, we discuss the potential problem of overtreatment with anti-PD-1 directed agents in general and introduce REFINE-Lung (NCT05085028), a UK multicentre phase 3 study of reduced frequency pembrolizumab in advanced non-small-cell lung cancer. REFINE-Lung uses a novel multi-arm multi-stage response over continuous interventions (MAMS-ROCI) design to determine the optimal dose frequency of pembrolizumab. Along with a similarly designed basket study of patients with renal cancer and melanoma, REFINE-Lung and the MAMS-ROCI design could contribute to practice-changing advances in patient care and form a template for future immunotherapy optimisation studies across cancer types and indications. This new trial design is applicable to many new or existing agents for which optimisation of dose, frequency, or duration of therapy is desirable.

## Introduction

Medical overtreatment and its negative effects on the health of individuals and societies has long been recognised.^1,2^ The history of cancer care is replete with examples of initial treatment excess evolving towards more rational, less intense use. Across diseases, a common pattern emerges of new treatments adopted with maximalist approaches to therapy, but with scarce evidence to support a relationship between therapeutic intensity and outcomes. This approach is followed by periods of critical evaluation based on concerns around the adverse consequences of overtreatment, including opportunity costs associated with the inefficient use of resources, quality of life, and toxicity. Justified by real-world clinical data and new biological insights, definitive optimisation trials across therapeutic modalities have reduced treatment intensity while preserving outcomes.^3–5^ A major obstacle in this process is the conduct of studies aiming to optimise treatment parameters including dose, schedule, and duration, given the inefficiencies of non-inferiority trial designs that are usually used.

A similar pattern of overuse and rationalisation is emerging in the field of cancer immunotherapy. In the last decade, a new class of antibody based, T-cell targeted immunotherapeutics have transformed cancer care across indications. Along with targeted small molecule inhibitors, these agents have brought in a new era of improved outcomes and rising costs of therapy.^6^ Overtreatment has potentially important effects on the quality of life of patients with cancer arising from high financial costs, increased hospital visits, and the potential for adverse events. Furthermore, as annual drug costs of over US$100 000 per patient are normalised globally,^7^ health-care systems are increasingly faced with the question of how cancer treatment can be afforded now and in the future.

This debate has accelerated calls to more effectively and rapidly optimise treatment regimens of new cancer drugs.^8^ The dose and schedule of multiple targeted agents have been subjected to scrutiny, resulting in post-licensing rationalisation of agents including abiraterone,^9^ ceritinib,^10^ and dasatinib.^11^ Regulatory bodies are increasingly focused on the issue of dose optimisation,^12^ with support from the US Food and Drug Administration (FDA) through Project Optimus that aims to promote, “a dose-finding and dose optimisation paradigm across oncology”.^13^ An immediate consequence is the FDA post-authorisation requirement that the novel KRAS-targeted drug sotorasib is tested at the approved dose of 960 mg versus 240 mg, on the basis of early-phase evidence of no relationship between dose and response in the range tested.^14^

Despite this growing consensus, a key limitation is that conventional non-inferiority trial designs do not typically evaluate treatment parameters (eg, dose, frequency, and duration) across a range of values. Here we discuss the mounting evidence of cancer immunotherapy over-treatment and its adverse effects. We present a novel trial design implemented in a large phase 3 lung cancer immunotherapy study that we propose could accelerate our advance towards rationally identifying optimal treatment regimens.

## Conventional early-phase trial designs are not suited for immunotherapies

Current concepts of early-phase trial design for cancer therapy were developed in the 1970s and 1980s during the era of cytotoxic chemotherapeutics, with data to support an expected positive relationship between the dose and the biological effect of these drugs. Consequently, early-phase trials have been designed to determine the maximum tolerated dose of new agents, defined as the highest dose that does not lead to severe short-term toxicity. Pharmacokinetic analysis has focused on measurements of blood distribution as a biomarker. Dose and administration schedules have subsequently been optimised to maximise drug delivery and availability.

But how should dose and regimen be selected for agents whose fundamental mechanism of action is poorly understood, without effective biomarkers to guide development? Monoclonal antibodies targeted to T-cell inhibitory receptors (checkpoint immunotherapies) are a case in point. These drugs were developed following the observation that naturally occurring immune responses can exert anti-cancer control but effectors are functionally impaired by the action of inhibitory receptors such as PD-1.

Checkpoint immunotherapies such as pembrolizumab, nivolumab, and cemiplimab target PD-1, while atezolizumab, avelumab, and durvalumab target the PD-1 ligand PD-L1 to enhance T-cell anti-cancer function—but their mechanism of action and optimal distribution characteristics are poorly understood. Thus, debate is ongoing about whether the target cell population are dysfunctional T cells,^15^ non-dysfunctional progenitor populations,^16^ or both. Similarly, checkpoint immunotherapies might act on T cells at the cancer site or enhance the activation and migration of T cells at distant sites such as draining lymph nodes. Clinical observations of responses and new toxicities observed long after treatment discontinuation highlight the potential of these drugs to exert effects even when they are presumably no longer active at the target receptor. Finally, even the relationship between PD-1 occupancy (the proportion of PD-1 molecules bound by the drug) and clinical outcomes is not established.

These biological uncertainties around the most relevant target population, site, and mechanism of action indicate the inadequacy of simple heuristics (eg, more is better) for determining optimal regimens for immunotherapies. Specifically, what biological parameter of drug effect should be chosen as a read-out to maximise? Since effective pharmacokinetic and dynamic biomarkers are unknown, trials with relevant clinical endpoints (eg, response or survival) are required to determine optimal parameters of dose administration, frequency, and duration.

## Clinical evidence of overtreatment with anti-PD-1 directed agents

Multiple lines of evidence suggest that current immunotherapy regimens can result in overtreatment.^17–19^ With conventional dose escalation approaches developed for the evaluation of cytotoxic drugs, early-phase studies of pembrolizumab and nivolumab sought, but failed, to identify a maximum tolerated dose for these agents, indicating no clear dose–response relationship. This was observed in the phase 1 KEYNOTE-001 study with doses of pembrolizumab ranging from 1 mg/kg to 10 mg/kg every 2 weeks^20^ and doses of nivolumab ranging between 0·1 mg/kg to 10 mg/kg every 2 weeks.^21^ This absence of a clinical dose–response relationship is reflected by measurements of PD-1 receptor occupancy. For nivolumab, receptor occupancy at 8 weeks did not differ substantially across the dose range (0·1–10·0 mg/kg).^21^ Crucially, although the drug was cleared from the circulation within days, occupancy reached a dose-independent plateau of 59–81% at over 8 weeks from a single infusion.^22^ Additional data are scarce, but in a 2022 report of five patients who discontinued nivolumab after long-term use, receptor occupancy varied from 40% to over 90% at between 20 weeks and 30 weeks after discontinuation.^23^ Although there are no published data directly measuring PD-1 receptor occupancy following pembrolizumab therapy, the effect of anti-PD1 on T-cell function was evaluated with an interleukin-2 release assay as part of a phase 1 study. This study showed little evidence of a dose–response relationship, particularly in the range of 1–10 mg/kg.^20^

Such findings are consistent with the high affinity of PD-1 inhibitors, yielding target saturation at low drug concentrations. For nivolumab, 0·04 μg/mL was sufficient to occupy over 70% of PD-1 molecules in vitro. This concentration was a third of the minimum serum-detectable level by enzyme linked immunosorbent assay, suggesting that conventional measurements of peak and trough serum drug concentrations might not be relevant markers to guide optimal dose and frequency of administration.

Although data from phase 1 trials of pembrolizumab show that target saturation is attained after one cycle with a dose of 0·1 mg/kg, drug distribution and receptor-occupancy modelling was done to establish the recommended phase 2 dose. These studies indicated that 2 mg/kg was required to attain 90% occupancy at drug-trough concentration within poorly vascularised tumour regions. This model makes a number of crucial assumptions: first, that pre-existing T cells infiltrating the tumour are the primary target of pembrolizumab, rather than circulating or lymph node resident cells; second, that the dynamics of drug clearance are constant; and finally that drug effects are transitory—ie, anti-cancer T cells exposed to pembrolizumab return to a baseline state of reduced functionality once PD-1 is no longer bound.

The notion that there is no relationship between dose and response in the range tested has been confirmed across multiple clinical trials. In the phase 1 KEYNOTE-001 study there was no evidence of a difference in response rate in patients with non-small-cell lung cancer (NSCLC) randomly assigned to pembrolizumab 2 mg/kg every 3 weeks or 10 mg/kg every 2 or 3 weeks in an exploratory analysis.^24^ Combined analysis of KEYNOTE trials (001, 002, and 003) similarly found no significant reduction in response rate at doses down to 1 mg/kg every 3 weeks.^25^ In larger studies, the phase 2/3 KEYNOTE-010 study found no significant difference in overall survival between patients with NSCLC randomly assigned to pembrolizumab 2 mg/kg versus 10 mg/kg.^26^ A similar absence of association between pembrolizumab dose and overall survival has been reported in melanoma and renal cell carcinoma trials with nivolumab doses between 0·3 and 10·0 mg/kg.^27,28^ This is supported by retrospective data showing that patients with NSCLC given flat dose pembrolizumab at 100 mg had equivalent survival outcomes to those given the 200 mg standard-of-care dose.^29^ Similar results have been reported with low-dose nivolumab (20 mg or 100 mg fixed dose *vs* 3 mg/kg every 2 weeks).^30^ Finally, patients given reduced-frequency pembrolizumab due to toxicity, non-toxicity related medical issues, or preference were not found to have compromised survival outcomes.^31^

Although there is no dose–response relationship in the tested range, there is evidence of a relationship between drug clearance and response.^32^ Re-analysis of data from KEYNOTE-002 and KEYNOTE-010 showed that slow pembrolizumab clearance after the first dose is associated with enhanced overall survival. Strikingly, this effect was independent of the dose delivered (2 mg/kg *vs* 10 mg/kg).^33^ Along with evidence that clearance declines over time in association with tumour stabilisation and metabolic normalisation,^34^ these data suggest that clearance itself is not implicated in tumour response but rather the importance of confounding factors such as cancer cachexia that could independently affect both the rate of antibody clearance and associate with patient outcomes.

In addition to dose and schedule, there is mounting evidence that prolonged durations of treatment might be unnecessary. Although pembrolizumab for NSCLC is licensed for up to 2 years, studies of patients who were responding to therapy and who stopped as planned at 2 years, or stopped earlier because of toxicity, show durable responses off treatment.^35–37^ In melanoma, similar evidence suggests that patients who stop treatment before progression because of toxicity or having achieved complete response have equivalent outcomes to those patients who continue treatment.^38,39^

These data have motivated several early stopping trials for patients with melanoma,^40–42^ but enthusiasm for such a trial in NSCLC was dampened by the results of the Checkmate 153 study. In this trial, the primary endpoint was the safety of nivolumab given every 2 weeks to patients older than 70 years with NSCLC and those with poor performance status. Additionally, an exploratory endpoint was included of efficacy among 163 patients randomly assigned at 1 year to stop or continue therapy for up to 2 years, with retreatment allowed at progression in the discontinuation group.^43^ Notably, patients in the continuous treatment group had a significantly better progression-free survival than did those in the 1-year fixed duration group (hazard ratio 0·42, 95% CI 0·25–0·71, median not reached). With 14·9 months’ follow-up, there was no evidence of a difference in overall survival between groups. Although these results suggest caution for future early stopping trials of immunotherapy in NSCLC, the study was not powered for overall survival. A further early-discontinuation trial of combined immunotherapy with nivolumab plus ipilimumab randomly assigned 265 patients with NSCLC to continue or stop treatment after 6 months of therapy.^44^ Although the study was terminated early and consequently underpowered, no difference in the primary endpoint of progression-free survival was observed.^44^ Consequently, although the question of whether early stopping of immunotherapy for patients with NSCLC is safe remains open, concerns remain around this strategy. Taken together, the high affinity, prolonged receptor occupancy and absence of a relationship between anti-PD-1 drug dose and clinical outcome across a wide range, suggest substantial scope for optimisation of dose and administration frequency.

## Optimising the use of immunotherapy

Arguments in favour of optimising immunotherapy administration regimens broadly centre on considerations of opportunity cost associated with inefficient resource use, quality of life, and toxicity.

Rising medication costs are a major cause for concern globally. In the USA, cancer care was estimated to cost $183 billion in 2015, and projected to rise to $246 billion in 2030, with similar trends worldwide.^45^ The financial cost of care places substantial pressures on the limited resources of health-care systems globally, with ongoing debate around how care should be funded and what compromises are necessary.^46,47^ From the perspective of the individual, high treatment cost has a negative effect on wellbeing, termed financial toxicity.^48^ This negative impact is particularly problematic in low-income and middle-income countries,^49,50^ where high costs form a barrier that excludes patients from accessing potentially life-changing agents, including immunotherapies.^51^ In some high-income countries, such as the UK, drugs that are considered insufficiently cost-effective are excluded from public funding, limiting access. Thus overtreatment in general is an inefficient use of health-care resources and optimised treatment regimens offer more patients opportunities to benefit. Some health-care economists have argued that the problem of high-care cost stems from how the pharmaceutical industry is incentivised; they suggest that a solution lies in the political domain, with approaches such as taxation and even nationalisation of pharmaceutical companies.^52–55^ Alternatively, more effective clinical-trial methods to find optimal regimens could represent a practical approach that solves the problem of overtreatment.

Checkpoint immunotherapies are among the most expensive medications to be routinely prescribed, with UK list prices of pembrolizumab approximately *£*90 000 and the cost of nivolumab about *£*70 000 for 1 year of treatment. Overall, efforts to optimise the dose, administration frequency, and duration could have global implications for health care in terms of increasing access to these agents and freeing resources for use on other health-care priorities.

At the patient level, beyond improving access to drugs, optimising the frequency of treatment administration will result in fewer hospital visits for treatment and pre-treatment evaluation. This reduction is expected to yield important enhancements in quality of life.^56^ In addition, optimised regimens could result in fewer immune-related toxicities, increasing the tolerability of treatment. Both anti-cancer and anti-normal tissue (autoimmune) effects of checkpoint immunotherapies are mediated by enhancing activation signals delivered downstream of the antigen sensing T-cell receptor.^57^ High affinity self-antigen reactive T-cell clones are targeted for elimination by thymic central tolerance mechanisms and self-reactive clones in the periphery are usually of low affinity. In contrast, high-affinity anti-cancer T cells recognising cancer specific antigens can escape thymic deletion and are found within tumours.^58,59^ Thus self-reactive and cancer-reactive populations could have different thresholds for activation. By reducing the activation of self-reactive clones to below a threshold for autoimmunity and preserving anti-cancer activity, optimised immunotherapy regimens could yield important safety benefits. These benefits could include reducing chronic immune related adverse events.^60^

## Determining the optimal frequency of immunotherapy administration

Regulatory limitations restrict the sharing of single dose drug vials between multiple patients.^61^ As a result, in the absence of pharmaceutical industry support, reduced dose immunotherapy studies are limited in practical terms, and optimisation of administration frequency is an attractive alternative. Optimised administration frequency has additional benefits in reduced hospital visits, further contributing to delivery of efficient care and yielding potentially important quality-of-life improvements.

But what is the optimal trial design to determine the lowest frequency of pembrolizumab administration that does not compromise efficacy? One option is a conventional two-arm non-inferiority trial comparing standard of care to a reduced-frequency intervention arm. Given the interest in reduced-frequency immunotherapy, multiple two-arm trials with this design are currently evaluating the performance of various extended intervals between doses ranging between 2-times and 4-times the standard-of-care interval (NCT04032418 and NCT04295863).^62^ But a major limitation of this design is that it requires investigators to guess the optimal alternative frequency to test, in the absence of preliminary data to guide rational selection of this alternative. In a conventional two-arm study, if the test frequency is poorly chosen, the trial will inevitably give a negative result even if another non-inferior frequency existed. If the study produced a positive result at the test frequency, whether longer frequencies than the tested one would also be non-inferior would remain unknown. A series of two-arm studies exploring multiple frequencies would be impractically time consuming and expensive.

Several novel approaches using Bayesian adaptive models exist to optimise continuous aspects of treatment such as dose in early-phase (1 and 2) trials.^63,64^ However, these are unsuitable for solving this issue in phase 3 studies of treatments already known to be effective and when the question of non-inferiority versus standard of care is crucial. These methods aim to find the maximum tolerated dose and balance this dose against efficacy, measured in a short timescale. Since early-phase studies of anti-PD1 directed agents have already demonstrated that the maximum tolerated dose is not reached in a 100-fold dose range of 0.1 mg/kg to 10.0 mg/kg, this design consideration is not relevant. Furthermore, since the allocation ratio between groups is altered according to the evaluation of short-term outcome data, these designs are poorly suited to cope with inherent features of late-phase trials (eg, the focus on long-term survival as the primary outcome). The only proposed alternative we are aware of is the DOOR/RADAR design,^65^ which involves categorising patients on the basis of benefits and harms into an overall clinical outcome and ranking them according to better outcomes or reduced treatment duration, or both. This design is being used in several trials^66–68^ but has been criticised^69^ for combining a clinical outcome with some aspect of treatment administration into a single variable that could hide important differences in the clinical outcome.

To address the need to explore a wide range of administration frequencies in a single, reasonably sized study, we have developed the multi-arm multi-stage response over continuous interventions (MAMS-ROCI) trial design. This design is an extension of our previous work to develop trial methods that could find the optimal duration of antibiotic therapy for a given infectious disease.^70^ The MAMS-ROCI design can in principle be used to establish the optimal value of any continuous treatment variable (ie, dose, duration, and frequency or schedule) and we focus here on administration frequency.

We propose that the optimal administration frequency can be found by randomly assigning patients to multiple treatment arms evenly distributed across a clinically reasonable range of frequencies. This design increases the probability of including the optimal arm in the study. Rather than comparing each test arm against the control 1:1, a model is fitted to estimate the frequency-response curve describing the relationship between frequency and efficacy across the entire range of alternatives tested. By sharing information across arms, the efficiency of the study is enhanced. With this model, the longest duration between doses with efficacy non-inferior to control 6-weekly therapy can then be determined. This design is thus capable of exploring a range of alternative frequencies when the optimal frequency is unknown, and often does so with a comparable number of patients to that of a conventional two-arm non-inferiority study.

## The reduced frequency pembrolizumab immunotherapy (REFINE-Lung) study

**Figure 1:**
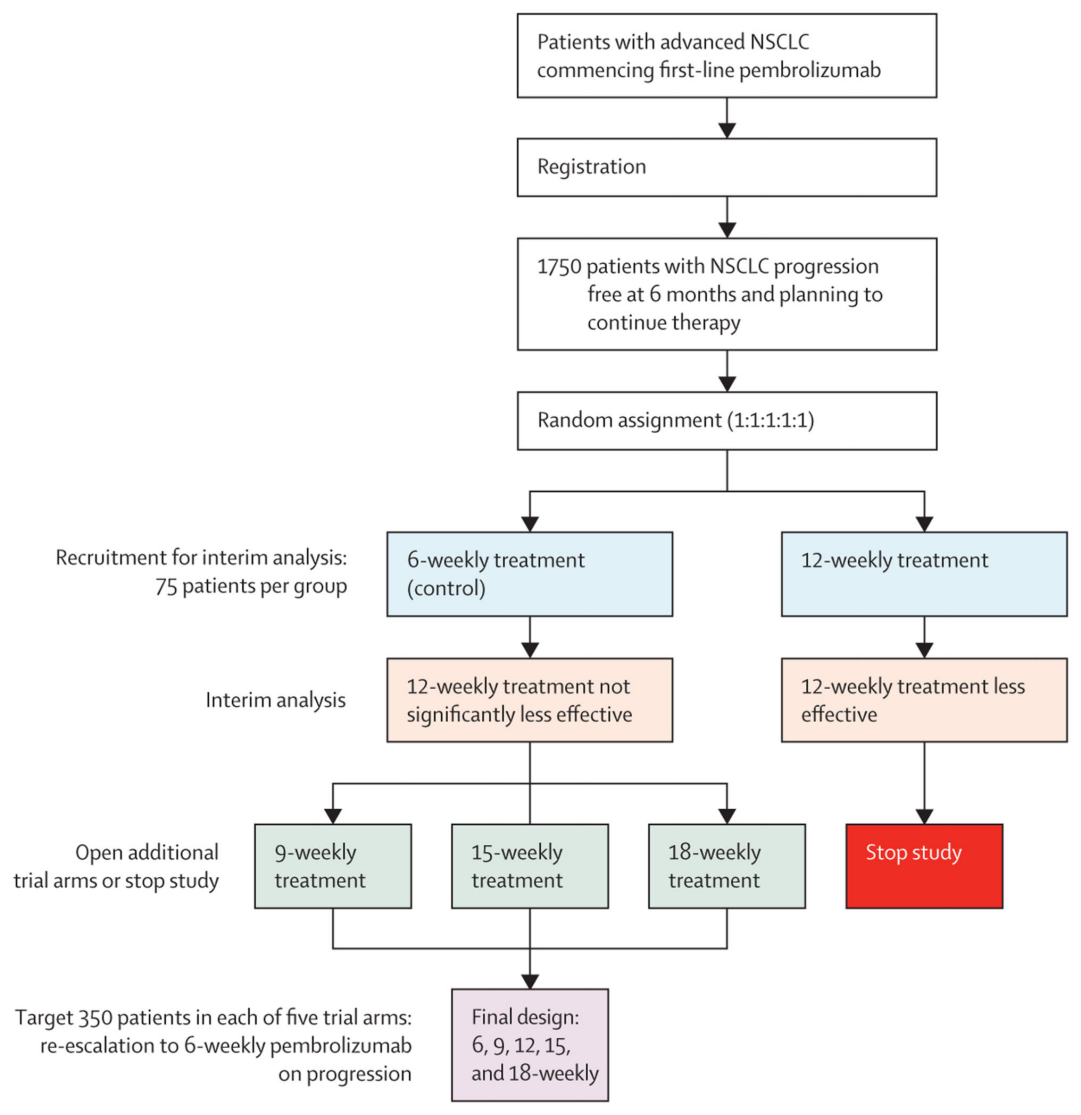
Reduced frequency pembrolizumab (REFINE-Lung) study flow chart NSCLC=non-small-cell lung cancer.

To find the optimally reduced frequency of first-line pembrolizumab (200 mg IV) for advanced NSCLC, REFINE-Lung will recruit 1750 patients who do not have progressive disease after 6 months of treatment and are planning to continue pembrolizumab therapy. In this pragmatic study aiming to maximise participation, patients with brain metastases and uncommon histologies are not excluded although patients on another clinical trial being treated with an investigational drug in addition to pembrolizumab are not eligible. Patients initially treated either with single-agent pembrolizumab or in combination with chemotherapy are eligible. Randomisation is evenly distributed between control (6-weekly pembrolizumab) or one of four frequency-reduced groups spaced at 3-weekly intervals (9, 12, 15, and 18-weekly arms; figure 1).

To mitigate the risk of needlessly exposing patients to reduced-frequency treatment that is potentially less effective than control, we will initially randomly assign patients to an internal pilot study comparing control 6-weekly versus 12-weekly therapy. If an event driven interim analysis does not show the 12-weekly treatment to be significantly less effective, subsequently recruited patients will also be randomly assigned to 9, 15, and 18-weekly treatment frequency groups. The primary outcome measure is overall survival at 2 years, with secondary outcome measures including quality of life, toxicity, and cost-effectiveness of the defined optimal dose frequency. Importantly, patients who develop progressive disease on a reduced-frequency arm will be offered treatment beyond progression with re-escalation to standard 6-weekly therapy.

## Practical design considerations for a MAMS-ROCI frequency optimisation study

Major practical issues to resolve include: (1) decisions around selecting the number and distribution of reduced frequency groups that patients are randomly assigned to; (2) how many patients are required; and (3) how to deal with the possibility that reduced frequency therapy could be detrimental. Through practical design considerations with reference to REFINE-Lung, in this Personal View we offer a guide for investigators interested in establishing similar trials including those optimising treatment variables other than administration frequency. Further details on design considerations for a MAMS-ROCI trial are published elsewhere.^71^

### Choice of trial arms

There is a clear rationale for the arm with the shortest duration between doses to be equivalent to standard of care (6-weekly in the context of pembrolizumab for lung cancer). In choosing the arm with the longest duration between doses we opted for an 18-weekly dose regimen for two reasons. First, phase 1 data had shown that a single dose of pembrolizumab was still bound to its PD-1 receptor target on immune cells with high occupancy after 140 days. Second, we established that the thoracic oncology health-care community and patients with lung cancer and their representatives were comfortable with this reduced frequency, knowing that in the event of disease progression treatment would be escalated back to the standard-of-care 6-weekly frequency.

**Figure 2:**
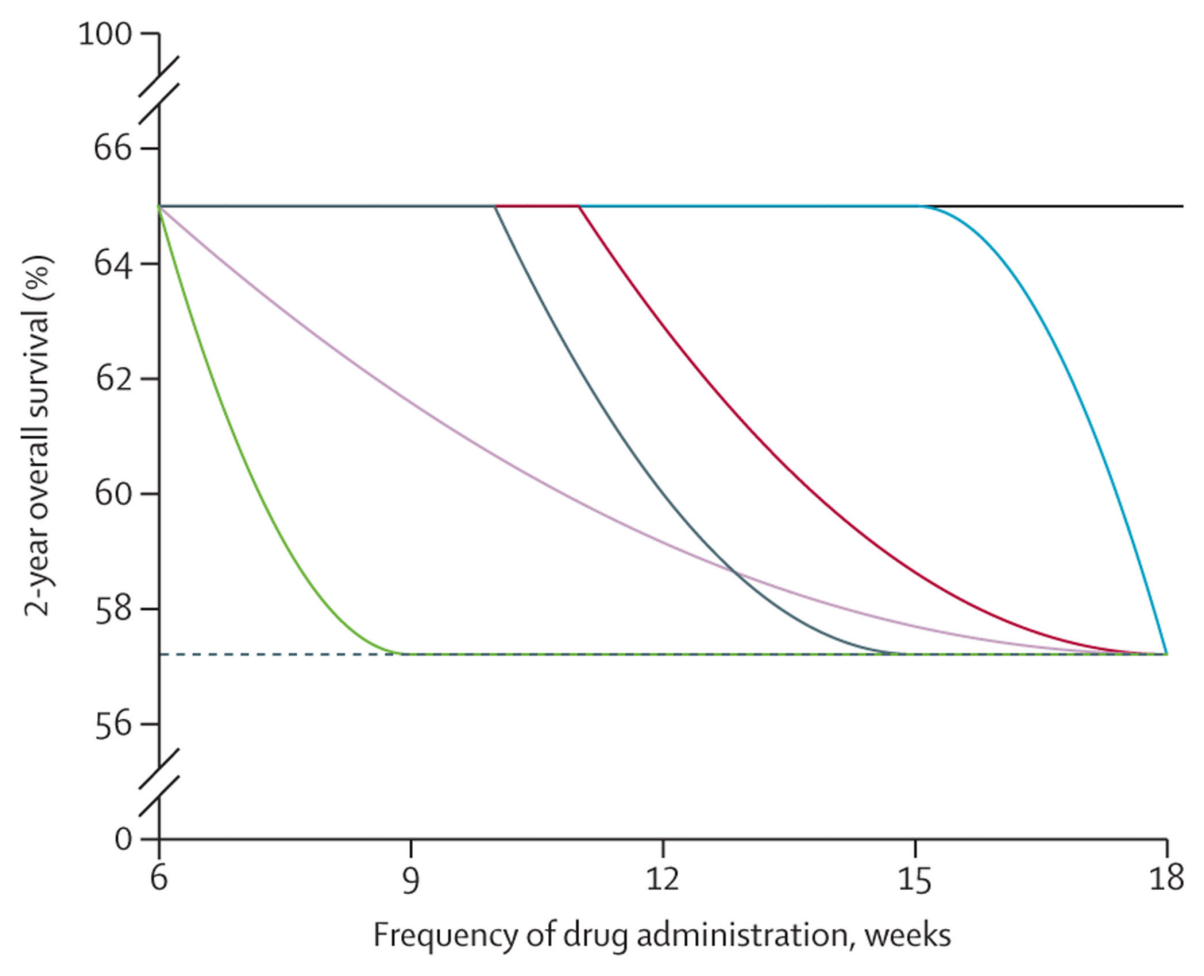
Type 1 error is controlled across a range of frequency–response scenarios We modelled the frequency–response relationship for various linear and non-linear scenarios, from a 2-year overall survival for 65% to 57·2% (the boundary of non-inferiority *vs* 6-week control). Each scenario is represented by a different line colour. In all cases (with 1550 patients) the type 1 error rate in a comparison of 18-week versus 6-week arms was under 5%.

The true frequency–response relationship cannot be assumed to be linear and could take various forms, thus complicating conventional approaches to sample size calculation, so we used simulation studies. To model the frequency–response relationship with simulated data, we applied a fractional polynomial regression approach with binary outcome data (overall survival at 2 years after commencing therapy). This strategy was found to be robust to a variety of possible frequency–response relationship curves, with type I error (the probability of finding no evidence of a difference between 18-weekly and 6-weekly arms where one exists) controlled across scenarios (figure 2).^72^

In additional simulation studies, we found that at least five trial arms are needed to model a likely range of relationships with the preferred analysis method with little benefit beyond seven trial arms. In general, optimal models were generated in experiments when intermediate trial arms are spaced approximately equidistantly. Since pembrolizumab is usually given 3-weekly or 6-weekly, we opted to retain 3-weekly intervals in the final design. Thus, a final design of 6, 9, 12, 15, and 18-weekly trial arms fitted the constraints defined earlier.

### Selecting the optimal administration frequency

**Figure 3:**
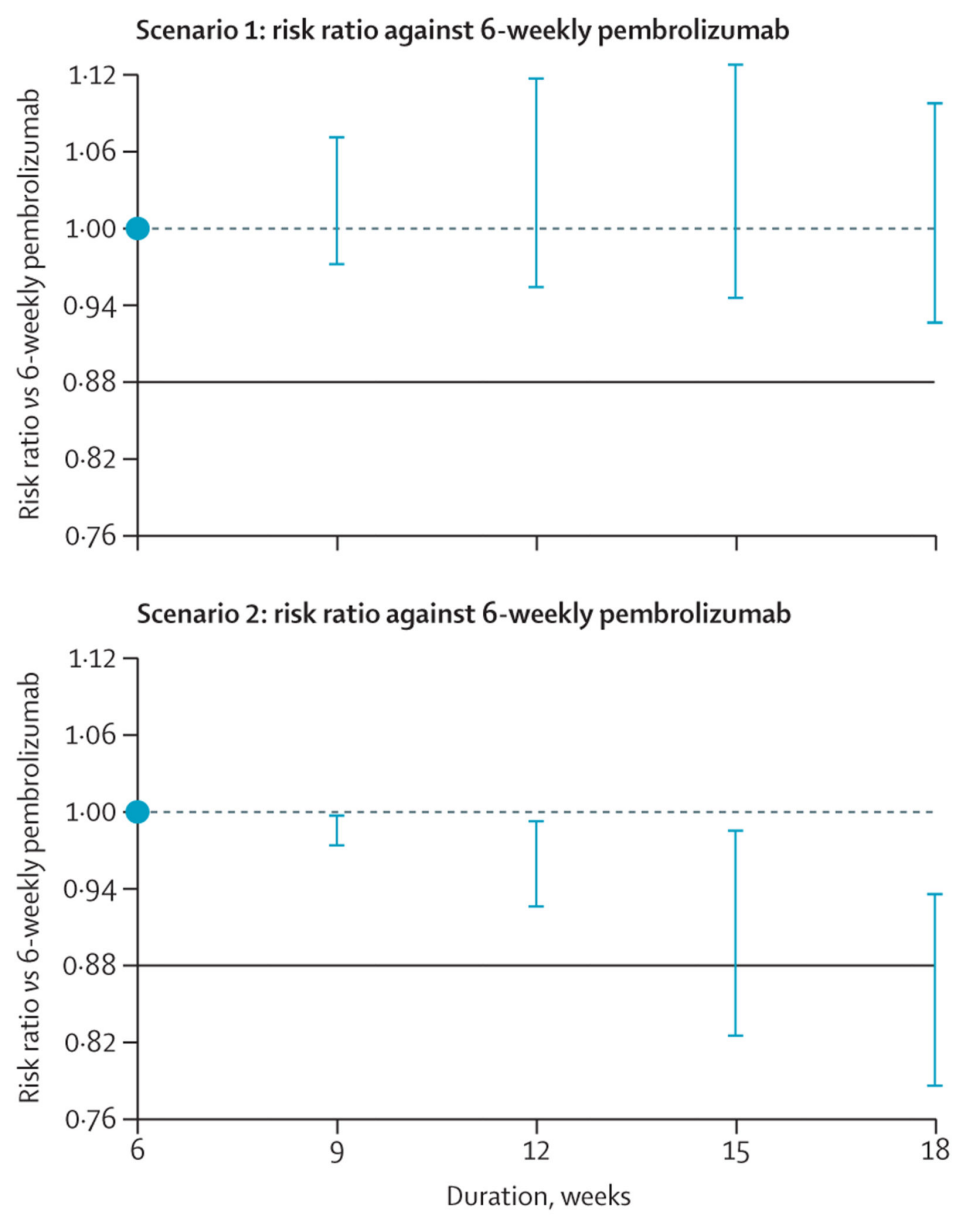
Illustration of how the optimal frequency-to-recommend will be determined Two possible frequency–response relationships are requested using simulated data. The blue horizonal lines represent the margin of non-inferiority versus 6-week control. In scenario 1, the 18-week arm is the longest frequency with a risk ratio within the non-inferiority margin. In scenario 2, the 12-week arm meets this criterion and is optimal.

The optimal administration frequency is defined prospectively as the least frequent dosing that is non-inferior to control 6-week therapy (figure 3). Practically, this means the lower boundary of the 95% CI around the risk ratio (RR) of the selected arm (RR defined as the experimental arm 2-year overall survival divided by the control arm 2-year overall survival) is above the non-inferiority margin. In line with multiple other studies,^73–75^ we prospectively defined the limit of non-inferiority in REFINE-Lung as preserving at least 50% of the effect of treatment versus control, yielding a RR margin of 0·88. Thus, an active arm can be declared non-inferior if the lower boundary of the 95% CI for the 2-year overall survival RR against 6-weekly is above 0·88. Once we have plotted the frequency–response curve, readers can input their own constraints to select the optimal frequency that they consider to be non-inferior to the standard 6-weekly regimen, although the trial may not be powered for that non-inferiority margin.

### Number of patients required

MAMS-ROCI also uses simulated data to determine the overall sample size required. In designing REFINE-Lung, this simulation assumes a 2-year overall survival of 65% based on available data from completed studies and also given that patients are enrolled in the study having already completed 6 months of treatment.^76–78^

For the sample size calculation, we generated data under the assumption that overall survival is the same irrespective of treatment frequency. On the basis of discussions with patient groups, this was felt to be an important starting point since it would not be ethical to randomly assign patients to treatment expected to be of suboptimal efficacy.

In each simulated trial, we randomly allocated each patient to one of the five study arms and randomly generated a binary outcome of overall survival at 2 years from a Bernoulli distribution. We then fitted a fractional polynomial regression model to estimate the frequency–response curve. The fitted model is used to show the RR and CI for each arm compared with the 6-weekly control.

**Figure 4:**
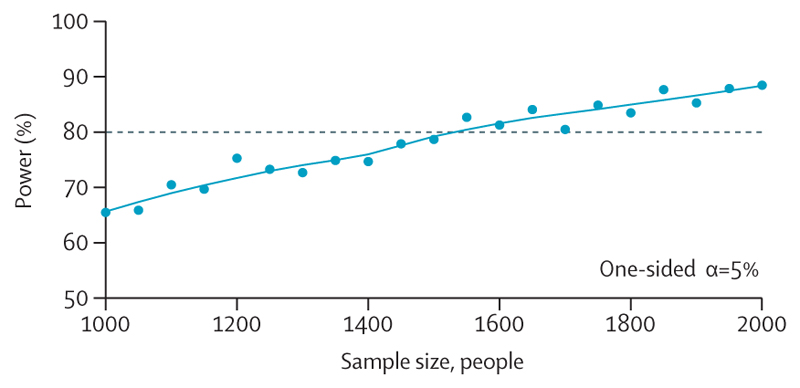
Sample size estimation Power was simulated across a range of sample sizes. 80% power is achieved with 1550 patients, with a one-sided alpha of 5%. Each point represents a simulated trial.

With these design parameters, equal randomisation of 1550 patients across five study arms was enough to achieve 80% power to find that the 18-weekly trial arm was non-inferior to the 6-weekly trial arm with a one-sided significance level of 5% (figure 4). Allowing for approximately 10% attrition (loss of patients from the study), the total sample size is 1750 patients.

It is instructive to compare this against a conventional non-inferiority design. In a standard two-arm study comparing 12-weekly versus 6-weekly therapy durations, assuming a 2-year overall survival of 65% and an 8% risk difference margin of non-inferiority (65%–0·88 × 65%), 1660 patients are required for 90% power and a 2·5% one-sided significance level allowing 10% attrition.

### Safety and reduced frequency of drug administration: an adaptive design element

Although we assume clinical equipoise, whether reduced frequency therapy could in general be detrimental is unknown. Thus, to open all four frequency-reduced trial arms simultaneously could be considered unethical. The MAMS-ROCI design tackles this issue by including an adaptive element. Individuals are initially randomly assigned to a standard-of-care 6-weekly group versus a 12-weekly group in the first stage of the study. The remaining study arms will open only if an interim analysis finds no significant difference in progression-free survival. In selecting the 12-weekly study arm as the comparator, we aimed to select the regimen with the longest duration between doses beyond which there would be little scope for optimisation. Thus if 12-weekly therapy was found to be detrimental, we would assume that the standard 6-weekly regimen cannot be meaningfully lengthened.

Although multiple stages could be considered to ensure study arms with less frequent dosing are opened only if those with more frequent dosing are found not to be inferior to control, this greatly complicates the trial design. Beyond the first stage, the independent data monitoring committee will review the data every 6 months to assess whether it is appropriate to continue randomisation to all study arms.

## Addressing overtreatment in oncology using the MAMS-ROCI design: dose, duration, and frequency optimisation

Broadening the scope beyond frequency optimisation of immunotherapy, the era of high-cost targeted cancer therapeutics has made it necessary for clinical trials to optimise their use of the drugs to bring benefits for patient health and quality of life. The academic community is well placed to address this need. The MAMS-ROCI design implemented in REFINE-Lung could be widely adopted to reduce guesswork inherent in current approaches to determining optimal dose, frequency of administration, and total duration of potentially toxic and expensive agents. Indeed, the novel MAMS-ROCI trial design serves as a new paradigm in testing dose-frequency reduction for immunotherapies across multiple cancer types. A basket trial approach across other cancer types is being established, with a cohort of patients with advanced renal cell carcinoma and melanoma currently open to recruitment (NCT04913025). The MAMS-ROCI design could have important benefits for patients and health-care systems globally by reducing inefficient overtreatment and improving the quality-of-care delivered, in addition to the patient-centred benefits of enhanced quality of life associated with fewer hospital attendances and reduced toxicity.

## Supplementary Material

Supp 1

## Search strategy and selection criteria

Searches of PubMed, the National Institute for Health and Care Excellence, the American Society of Clinical Oncology, and the European Society for Medical Oncology meeting abstracts and clinical trial databases were performed with combinations of the terms immunotherapy, pembrolizumab, nivolumab, lung, cancer, trials, pharmacokinetics, and pharmacodynamics between Jan 1, 2010, and Nov 1, 2022. Randomised phase 3 trials were considered to provide the best evidence.
