# Supplementary material for: REFINE-Lung implements a novel multi-arm randomised trial design to address possible immunotherapy overtreatment
Source: Lancet Oncol. Author manuscript; Available in PMC 2025 Feb 1. (PMC7617361; doi:10.1016/S1470-2045(23)00095-5)
Supplement: Supp 1 [file EMS202200-supplement-Supp_1.pdf]

THE LANCET
Oncology

Supplementary appendix

This appendix formed part of the original submission and has been peer reviewed. We post it as supplied by the authors.

Supplement to: Ghorani E, Quartagno M, Blackhall F, et al. REFINE-Lung implements a novel multi-arm randomised trial design to address possible immunotherapy overtreatment. *Lancet Oncol* 2023; **24:** e219–27.

Appendix

The REFINE-Lung Investigators group (RLIG)

RLIG consists of; Charing Cross Hospital: Michael Seckl, Ehsan Ghorani, Joanne Evans, Pollyana D'Avila Leite, Sanjary Mistry, Emily White and Amalia Saucan; The Royal Marsden Hospital: Mary O'Brien, Sanjay Popat, Charlotte Milner-Watts, Libby Hennessy, Bianca Rock, Jaishree Bhosle, Anna Minchom, Nadza Tokaca and Hazel O'Sullivan; The Christie Hospital: Fiona Blackhall, Fabio Gomes, Kate Brown, Stephen Boyd and Laura Moliner Jimenez; Peterborough City Hospital: Sarah Treece, Elisa Barter, Kerrie Cavanagh, Terri-Anne Baker, Abigail Hollingdale and Sudipta Datta; Nottingham City Hospital: Jason Adhikaree, Rebecca Ashton and Karen Newcombe; Queen's Hospital: Kathryn Tarver, Simon Ball, Jonathan Shamash, Neale O'Brien and Thi Vu; Leeds Hospital: Pooja Jain, Amy Humphries, Gwendolyn Saalmink and Liz Ashford; Royal Cornwall Hospital: Grant Stewart, Toby Talbot and Jane Broom; NHS Lothian: Colin Barrie and Lisa Thomson.
